# Sources of Heavy Metals and Their Effects on Distribution at the Sediment–Water Interface of the Yellow Sea Shelf off Northern Jiangsu

**DOI:** 10.3390/toxics14020133

**Published:** 2026-01-29

**Authors:** Wenyu Liu, Yu Li, Xinjun Wang, Yuhan Cao

**Affiliations:** School of Marine Technology and Geomatics, Jiangsu Ocean University, Lianyungang 222005, China; 2024220222@jou.edu.cn (W.L.); liyu241@sina.com (Y.L.); 2023220224@jou.edu.cn (X.W.)

**Keywords:** sediment–water interface, heavy metals, principal component analysis (PCA), random forest (RF), positive matrix factorization (PMF), source-specific partition coefficient (S-Kp)

## Abstract

To investigate the distribution, sources, and partitioning of heavy metals at the sediment–water interface in the northern Jiangsu coastal waters, seawater and sediment samples were collected from 24 stations east of Yanwei Port in April 2021. The concentrations of seven heavy metals (Cu, Pb, Zn, Cd, Cr, Hg, and As) and environmental parameters were determined. Methods including principal component analysis (PCA), random forest (RF), positive matrix factorization (PMF), the partition coefficient (Kp), and the source-specific partition coefficient (S-Kp) were applied. The results showed the following: (1) The overall concentration order was Zn > Cu > As > Pb > Cd > Hg in seawater and Zn > Cr > Cu > Pb > As > Hg > Cd in sediments, with Cd and Pb characterized by high spatial variability. (2) PCA and RF indicated that dissolved heavy metals were mainly influenced by dissolved oxygen, petroleum, phosphate, and dissolved inorganic nitrogen, with DIN playing a common dominant role. PMF revealed three main sources for sediment metals: agricultural (contributing notably to Cu and Zn), traffic and industrial exhaust (dominating Pb, Cr, and Hg inputs), and industrial (primarily affecting Cd, Cr, and Pb). (3) Kp analysis suggested that Pb, As, and Cu were readily adsorbed by sediments, while Cd, Hg, and Zn tended to remain dissolved. Critically, S-Kp demonstrated source dependent partitioning: Pb derived from industrial sources was almost entirely associated with sediments, while Cu and Zn originating from traffic and industrial exhaust emissions were predominantly present in the aqueous phase, and Cu and Pb derived from agricultural sources were largely deposited in sediments. These findings provide a scientific basis for heavy metal pollution control in the region.

## 1. Introduction

With the acceleration of industrialization, urbanization, and agricultural expansion, large quantities of heavy metals enter the marine environment through industrial emissions, agricultural runoff, traffic exhaust deposition, and other pathways, posing potential threats to marine ecosystem stability and human health [1,2]. Heavy metals are characterized by non-degradability, easy accumulation, and transmission along the food chain. Their migration and transformation processes at the water–sediment interface directly determine the ecological risks of pollution [3,4,5]. Sediments serve as a “sink” for heavy metals, but under changing environmental conditions, they may also become a secondary pollution “source” via desorption. Thus, analyzing the distribution characteristics, sources, and partitioning behavior of heavy metals at the sediment–water interface is a core scientific issue in marine pollution prevention and control.

The northern Jiangsu coastal waters, situated in the western Yellow Sea adjacent to important ports and industrial clusters (e.g., Yanwei Port and Lianyungang Port), are affected by agricultural non-point source inputs from the north Jiangsu plain, making it a typical area under the combined influences of industry, agriculture, and shipping [6]. As an important aquaculture base and ecological protection zone along China’s eastern coast, the water environmental quality of this region is closely related to regional economic development and ecological security [6]. In recent years, with the rapid development of chemical, metallurgical, shipbuilding, and other industries in northern Jiangsu, as well as the widespread use of chemical fertilizers and pesticides in farmland, heavy metal input loads have continued to increase, leading to accumulation in some local waters [7]. Previous studies have mostly focused on heavy metal pollution in a single medium, and systematic research on heavy metals at the sediment–water interface in the northern Jiangsu coastal waters remains scarce [8]. In particular, integrated analysis of the “source-partitioning-risk” chain is lacking, posing challenges for precise pollution control in this region.

Currently, heavy metal source apportionment techniques have evolved from single correlation analysis to multi-model coupling applications. Principal component analysis (PCA) can identify associations between heavy metals and environmental factors through dimensionality reduction, providing preliminary clues for pollution source classification [9,10,11]. The positive matrix factorization (PMF) model can quantitatively resolve the contribution ratios of various pollution sources under non-negative constraints, and their combination substantially enhances the reliability of source apportionment outcomes [12,13,14]. In addition, the partition coefficient (Kp) is a key parameter describing heavy metal migration at the water–sediment interface [15,16], while the source-specific partition coefficient (S-Kp) can further quantify the partitioning differences in heavy metals from different sources, offering a new perspective for revealing the regulatory mechanisms of pollution sources on interfacial behavior [17].

Based on measured data of seven heavy metals (Cu, Pb, Zn, Cr, Cd, Hg, and As) in water and sediment from the northern Jiangsu coastal waters in spring 2021, this paper analyzes the occurrence of heavy metals in different media, explores their distribution characteristics and main sources at the water–sediment interface, and elaborates on the partitioning relationships of heavy metals from different pollution sources between water and sediment. The research findings can provide a scientific basis for heavy metal pollution prevention and control in the coastal waters of Lianyungang and Yancheng.

This study investigates the influence of environmental factors on heavy metal concentrations in the northern coastal waters of Jiangsu Province, identifies the sources of heavy metal pollution in sediments, and examines the distribution and partitioning behavior of heavy metals at the sediment–water interface. Multiple methods (PCA, RF, PMF, S-Kp, etc.) were employed to comprehensively assess the pollution status of the northern coastal waters of Jiangsu from multiple perspectives.

## 2. Materials and Methods

### 2.1. Study Area and Sample Collection

**Figure 1:**
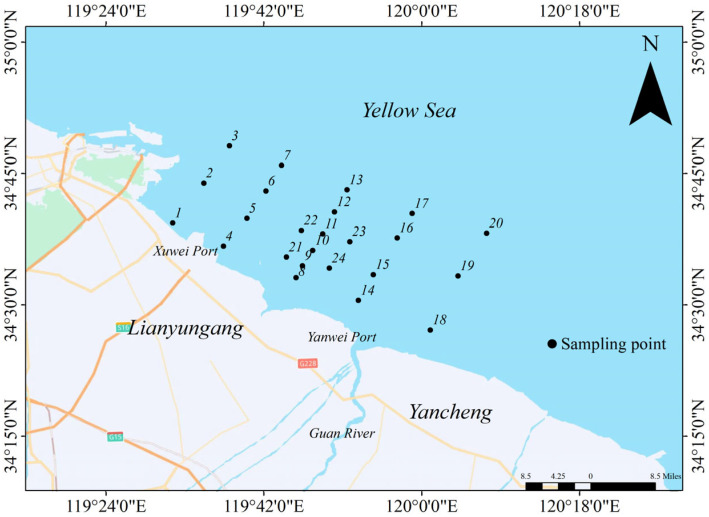
Layout diagram of the survey stations. Green for forests, white for land, blue for oceans, orange for highways, and yellow for ordinary roads. Black dots indicate sampling positions and sequence.

This study was conducted in the coastal waters east of Yanwei Port in April 2021. A total of 24 sampling sites were set in the coastal waters east of Yanwei Port (Figure 1). Mid-layer water samples were collected at all sites, with surface sediment samples (0–5 cm) obtained at 12 of them. Water depths at the sampling sites ranged from 5.2 to 18.7 m, with an average of 11.3 m. Mid-layer water samples (0.6 H, where H is water depth) were collected using an organic glass water sampler, and surface sediments were collected using a grab sampler. In all stations, water depth and salinity were recorded. The monitoring parameters included the contents of heavy metals [copper (Cu), lead (Pb), zinc (Zn), cadmium (Cd), chromium (Cr), mercury (Hg), and arsenic (As)] in water and sediment as well as other chemical indices [dissolved inorganic nitrogen (DIN), phosphate phosphorus (PO_4_-P), dissolved oxygen (DO), pH, chemical oxygen demand (COD), sulfide (S), and oil content]. For the determination of heavy metals in seawater, a preconcentration step was performed using chelating resin (Dowex XAD-8, manufactured by The Dow Chemical Company, Midland, MI, USA) prior to ICP-MS analysis to improve detection sensitivity. Sediment samples were subjected to total digestion with a mixture of HNO_3_-HCl-HF (volume ratio 3:1:1) using a microwave digestion system (CEM MARS 6, manufactured by CEM Corporation, Matthews, NC, USA) to ensure complete dissolution of heavy metals. The particle size analysis of the samples was performed using a laser particle size analyzer (Malvern Mastersizer 3000, manufactured by Malvern Panalytical Ltd., Malvern, Worcestershire, UK). Prior to testing, organic matter (added with 30% H_2_O_2_) and carbonate (added with 10% HCl) were removed from the samples. According to the Udden–Wentworth particle size classification standard, the samples were divided into three particle sizes: sand (>63 μm), silt (4–63 μm), and clay (<4 μm). Measurements of salinity and pH were conducted at all three water layers (surface, mid-layer, and near-bottom) using a portable pH-DO-Salinity meter (Model: YSI ProPlus). All sampling, storage, and transportation procedures followed the Specifications for Oceanographic Monitoring [18].

### 2.2. Sample Analysis and Quality Control

The analysis of samples in this study followed national standards and industry specifications. The specific methods are as follows: Nitrite, nitrate, ammonia, and PO_4_-P were determined using flow analysis according to HY/T 147.1-2013 [19]. COD was determined according to GB 17378.3-2007 using the potassium dichromate oxidation method, which is applicable for seawater samples after adjusting for chloride interference with mercuric sulfate [18]. Cu, Zn, Cr, Cd, Pb, and As were determined by inductively coupled plasma mass spectrometry (ICP-MS) based on HY/T 147.2-2013 [20]; Hg was determined by atomic fluorescence spectrometry; sulfide was analyzed by methylene blue spectrophotometry; oil content and suspended solids were determined by ultraviolet spectrophotometry and gravimetry, respectively, with all following GB 17378.5-2007 [21]. pH, temperature, and salinity were measured directly with a portable pH-DO-Salinity meter (Model: YSI ProPlus), which was calibrated before use according to the manufacturer’s instructions. All analytical procedures strictly adhered to standard operating protocols, with analytical errors controlled within 15% to ensure data accuracy and comparability.

### 2.3. Principal Component Analysis (PCA)

Principal component analysis (PCA) can reflect the information from the original data using a few indicators and is widely applied in heavy metal source studies. PCA reduces the dimensionality of original data by orthogonally rotating initial factor loadings, condenses the original information into fewer new variables, and then identifies the main factors based on the factor loadings of each variable [9,10,11]. Principal component analysis was performed using SPSS 25 (manufactured by IBM Corporation, Armonk, NY, USA).

### 2.4. Positive Matrix Factorization (PMF)

Positive matrix factorization (PMF) is used to identify pollutant sources. In PMF, the sample concentration data matrix (X) is decomposed into a factor contribution matrix (G) and a factor profile matrix (F) under non-negative constraints [22,23]. The sources can be determined based on the decomposition results, allowing relevant pollution information to be collected [24]. The PMF model is expressed as follows:

(1) $$x_{ji}=\sum_{p=1}^{k}g_{jp}f_{pi}+e_{ji}$$

(2) $$f_{p}=\frac{\sum_{i=1}^{m}f_{ip}}{m}$$

where **x***_ji_* represents the concentration of heavy metal *i* in sample *j*, *g_jp_* represents the contribution of factor p in sample *j*, *f_pi_* represents the proportion of factor *p* in heavy metal *i*, *f_p_* represents the proportion of factor *p* across heavy metals, *m* is the number of heavy metals, *e_ji_* represents the modeling error for the concentration of heavy metal *i* in sample *j*, and *k* is the number of factors. This study employed EPA PMF 5.0 (US Environmental Protection Agency) to perform PMF analysis on the data.

### 2.5. Random Forest (RF)

Random forest (RF), an ensemble learning algorithm developed by Breiman, is built on the Bagging (Bootstrap Aggregating) framework. By integrating multiple decision trees, it enhances model stability and generalization capabilities. It is widely used in environmental science for identifying pollutant distribution drivers and source apportionment [25]. RF was employed in this study to assess the relative importance of environmental factors in determining heavy metal concentrations in water bodies. For regression analysis, RF outputs the average prediction value of all decision trees:

(3) $$\hat{y}=\frac{1}{T}\sum_{t=1}^{T}f_{t}(x)$$

where *T* denotes the number of trees, while *f_t_*(*x*) represents the predicted value of the input feature vector, *x*, by the *T*-th tree. The model employs the mean decrease in MSE as the metric for the feature importance assessment, where higher scores indicate a greater influence of environmental factors on heavy metal distribution. We use the sklearn library in PyCharm 2024.2.3 for modeling.

### 2.6. Source-Specific Partition Coefficient (S-Kp)

The partition coefficient (*Kp*) is used to analyze the distribution of heavy metals in water and sediment. It is defined as the ratio of the average heavy metal concentration in sediment to that in water [26,27], as expressed in the following formula:

(4) $$Kp=\frac{C_{s}}{C_{w}}$$

where *C_s_* and *C_w_* are the average concentrations of the heavy metal in sediment and water, respectively. Combining the source apportionment results with *Kp*, the source-specific partition coefficient (*S-Kp*) [17] was calculated to study the distribution of heavy metals from different sources in the environment. The formula is as follows:

(5) $$S\text{-}Kp_{i}=\frac{C_{s,i}}{C_{w,i}}$$

where *S-Kp_i_* is the source-specific partition coefficient for factor *i*, *C_s_*_,*i*_ is the concentration of the heavy metal from source *i* in sediment, and *C_w_*_,*i*_ is the concentration of the heavy metal from source *i* in water.

## 3. Results and Discussion

### 3.1. Distribution Characteristics of Water Environmental Factors

**Figure 2:**
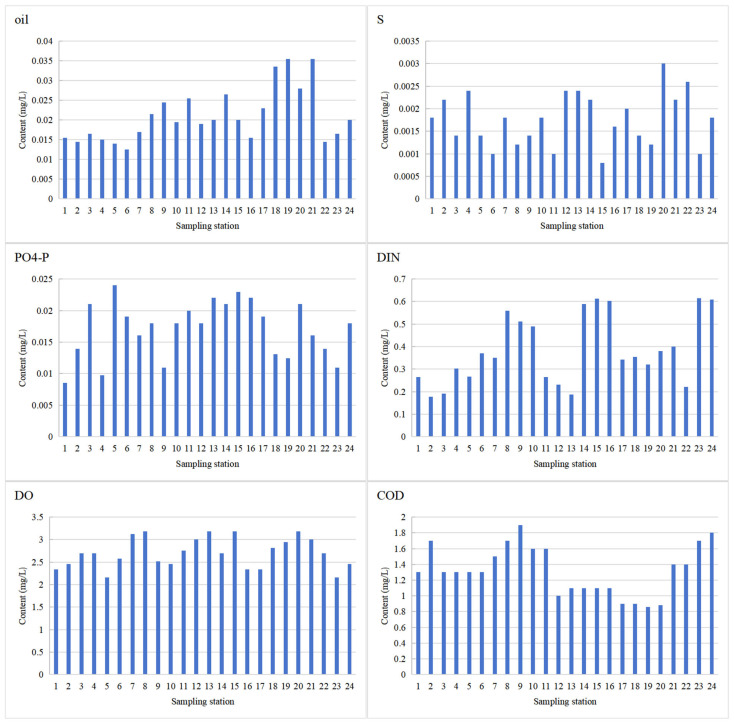
Concentrations of aquatic environmental factors at coastal waters of the northern part of Jiangsu Province (Yellow Sea).

The waters in northern Jiangsu were generally weakly alkaline, with pH values ranging from 8.0 to 8.5. All environmental factors (oil, S, PO_4_-P, DIN, DO, and COD) showed significant spatial heterogeneity across the 24 sampling sites (Figure 2), with different driving mechanisms for each factor. Oil concentration exhibited obvious peaks at some sampling sites while remaining relatively stable at others, indicating intermittent oil pollution inputs potentially associated with time-dependent human activities (e.g., shipping and industrial discharge) [28]. Sulfide (S) concentration peaked at Site 20, with a few other sites also showing relatively high values. Sulfide is closely associated with microbial decomposition processes under anaerobic conditions, and its fluctuations reflect the dynamic changes in water DO levels and organic matter degradation [7]. PO_4_-P concentration showed high values at multiple sites with frequent overall fluctuations. DIN concentration was highest near the Guan River estuary. DIN is a core indicator of nitrogen pollution, with high-value areas directly related to nitrogen source inputs (sewage and agricultural runoff) and nutrient demands for algal growth [29]. DO and COD levels were generally low overall, reflecting water aeration and biological oxygen consumption intensity. This reflects a reduced capacity of water bodies to mitigate pollution [30].

### 3.2. Occurrence and Spatial Distribution Characteristics of Heavy Metals in Water and Sediment

#### 3.2.1. Occurrence and Spatial Distribution Characteristics of Heavy Metals in Water

**Figure 3:**
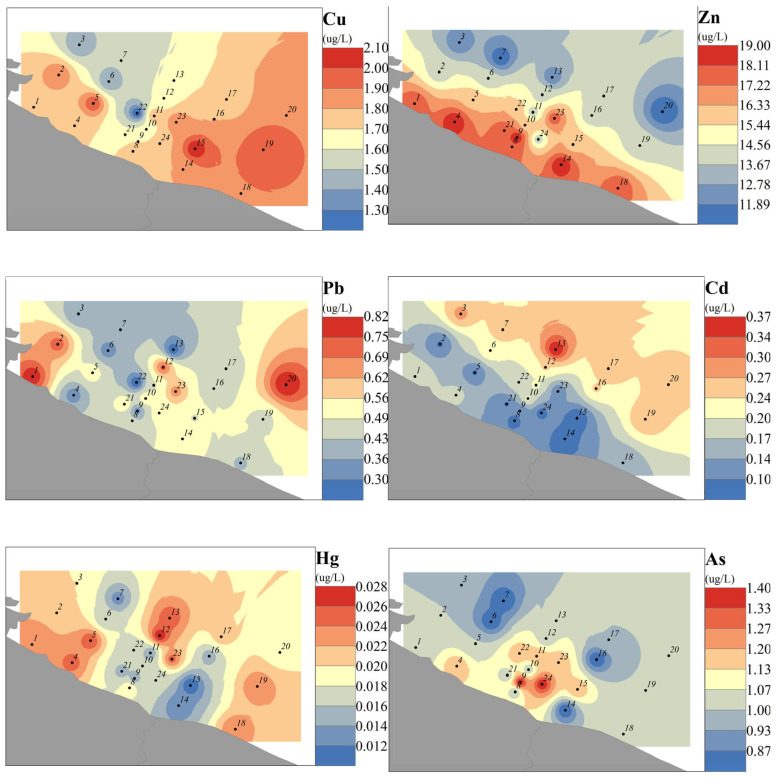
Distribution of heavy metals in the waters of the northern coast of Jiangsu Province (Yellow Sea). Gray indicates land, and numbers represent sampling locations and order.

The heavy metal contents in water samples from the 24 sites in the study area overall complied with the Class III seawater quality standards of the seawater quality standard (GB3097-1997) [31]. Figure 3 shows the spatial distribution of heavy metals at each site, exhibiting the characteristic order of Zn > Cu > As > Pb > Cd > Hg (Table 1). Chromium (Cr) was not detected. The mean concentrations were as follows: Cu: 1.73 µg/L, Pb: 0.49 µg/L, Zn: 15.38 µg/L, Cd: 0.18 µg/L, Hg: 0.019 µg/L, and As: 1.04 µg/L. The terrestrial input is depicted in Figure 3 and is mainly for Cu, Zn, Hg, and As, while for Cd and Pb, the spatial distribution indicates higher levels further away from the coast. High concentrations of Pb in seawater were mainly influenced by shipping and atmospheric deposition [7]. The lowest concentration of Cd exceeded the Class II standard of China’s seawater quality standards. The spatial distribution of heavy metals in seawater confirms that terrestrial pollution input is the primary driver of heavy metal accumulation in this region.

The coefficient of variation (CV) for each heavy metal in the study area seawater ranged from 12.1% to 43.9%. Cd and Pb had CVs of 43.9% and 31.9%, respectively, classifying them as elements with high spatial variability (CV ≥ 30%) and reflecting localized pollution and marked spatial heterogeneity. Hg had a CV of 26.1%, classifying it as an element with moderate spatial variability (20% ≤ CV ≤ 30%), with occasional outliers at individual sites. Zn, As, and Cu had CVs of 16.3%, 15.6%, and 12.1%, respectively, classifying them as elements with low spatial variability (CV ≤ 20%) and indicating a relatively uniform distribution in seawater with small spatial differences.

An ecological risk assessment based on the Risk Quotient (RQ) was conducted on heavy metals in water. Using the Class II seawater-quality heavy metal standards from the seawater quality standard (GB3097-1997) as background values, the RQ for each site was calculated [32] using the formula RQ = MEC/PNEC, where MEC is the measured environmental concentration and PNEC represents the corresponding environmental quality standard. RQ < 0.1 indicates a low ecological risk; 0.1 ≤ RQ < 1.0 indicates a moderate ecological risk; RQ ≥ 1.0 indicates a high ecological risk. The results showed that RQ values at all sites fell within the moderate ecological risk range (0.1 ≤ RQ < 1.0), indicating an overall low level of pollution in the study area. Cu and Zn posed a moderate ecological risk at all sites, whereas Hg and Pb posed a moderate risk at 12 and 10 sites, respectively. Cd and As posed a low ecological risk across all sites.

A comparison of heavy metal risk levels in the seawater between the study area and other domestic and international coastal regions (Table 2) shows that concentrations in the study area are significantly lower than those in industrialized regions such as the Pearl River Estuary [33] and heavily polluted waters such as the Persian Gulf [34] while being comparable to levels reported for ecologically protected areas such as Chesapeake Bay, USA [35]. Similar heavy metal pollution levels have been reported in the coastal waters of Korea [36]. This suggests that heavy metal pollution in the northern Jiangsu waters is at a low level, with relatively low associated ecological risks.

#### 3.2.2. Occurrence and Spatial Distribution Characteristics of Heavy Metals in Sediment

**Figure 4:**
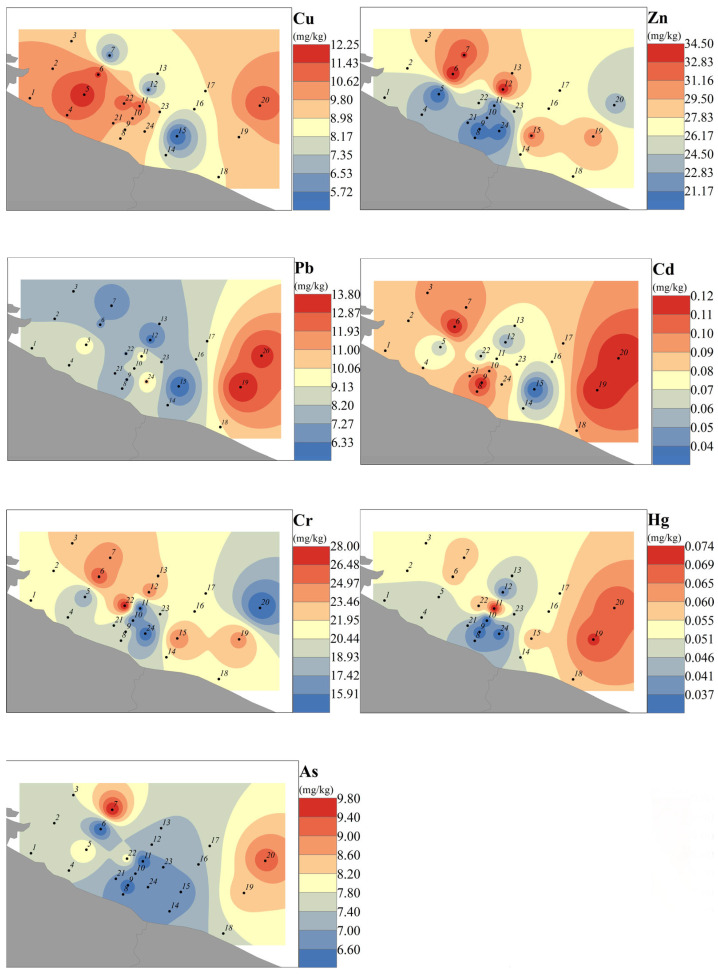
Distribution of heavy metals in sediments of the northern coastal waters of Jiangsu Province (Yellow Sea). Gray indicates land, and numbers represent sampling locations and order.

The overall level of heavy metals in sediments within the study area was low, with concentrations at all sites below the Class I sediment quality standards of the Marine Sediment Quality (GB18668-2002) [37]. Figure 4 illustrates the spatial distribution of heavy metals in sediments at each site, exhibiting the overall characteristic order of Zn > Cr > Cu > Pb > As > Hg > Cd (Table 3). The mean concentrations were as follows: Cu: 9.42 mg/kg, Zn: 26.25 mg/kg, Pb: 8.75 mg/kg, Cd: 0.09 mg/kg, Cr: 20.8 mg/kg, Hg: 0.0525 mg/kg, and As: 7.5 mg/kg. High-concentration sites for Cu, Cr, Zn, and Hg were located near the coast. The sedimentary grain size distribution is predominantly composed of silt, accounting for 65.2% (52.8–78.4%) on average, followed by sand particles at 28.5% (16.3–41.2%) and clay particles at 6.3% (3.5–9.7%). Correlation analysis reveals a significant positive correlation (r = 0.52–0.68, *p* < 0.05) between heavy metal concentrations (Cu, Zn, Pb, and Cr) and the clay and silt fractions, while showing a negative correlation (r = −0.45–0.59, *p* < 0.05) with the sand fraction. These results indicate that grain size distribution is a key factor influencing the spatial distribution of heavy metals in the study area. Furthermore, Cu, Pb, Cd, Hg, and As showed higher concentrations in the eastern part of the study area, which is near the estuary where treated wastewater is discharged from the Lianyungang Chemical Industrial Park and the Yancheng Binhai industrial zone on the west side [8]. These spatial distributions indicate that sedimentary heavy metals are mainly derived from anthropogenic and industrial activities.

The CV for each heavy metal in the study area sediment ranged from 15.5% to 29.4%. Pb, Cd, Hg, Cu, Cr, and Zn had CVs of 29.4%, 28.6%, 27.6%, 24.2%, 23.3%, and 22.2%, respectively, classifying them as elements with moderate spatial variability (20% ≤ CV ≤ 30%) with relatively significant spatial differences, as they are locally influenced by human activities. As had a CV of 15.5%, classifying it as an element with low spatial variability (CV ≤ 20%) with a relatively uniform spatial distribution and small spatial differences. To better understand the pollution degree of heavy metals in sediment relative to the natural environment, the geo-accumulation index (Igeo) [38] was used for assessment. The formula is Igeo = log_2_(Cs/1.5Bs), where Cs is the measured content of heavy metal s in sediment, while Bs is the background concentration of heavy metal s. Igeo < 0 indicates no pollution; 0 < Igeo ≤ 1 indicates slight pollution; 1 < Igeo ≤ 2 indicates moderate pollution; Igeo > 2 indicates severe pollution. The adopted background value of heavy metals is the global crustal background value, with Cu: 25 mg/kg, Zn: 65 mg/kg, Pb: 14.8 mg/kg, Cd: 0.10 mg/kg, Cr: 126 mg/kg, Hg: 0.04 mg/kg, and As: 1.7 mg/kg [39]. The results showed that among the sediments in the study area, only As was polluted, mainly slightly to moderately, while other heavy metals did not exceed the enrichment level of background values and were in an unpolluted state.

Comparing sediment heavy metal risk levels in the study area with similar domestic and international waters (excluding Hg and As), relative to the East China Sea area, only the Pb content is slightly higher (Table 4); other heavy metals are at medium levels, and the overall pollution degree is comparable [40]. Compared to the Mediterranean coastal area, the maximum values of Cu and Zn in the study area are lower than their minimum values there; only Pb pollution is relatively elevated, while pollution levels for other heavy metals are low [41]. Relative to Indian coastal waters, all heavy metal contents in the study area are at low levels, demonstrating a clear advantage in terms of ecological risk [42].

### 3.3. Source Apportionment and Contribution Rates of Heavy Metals

#### 3.3.1. Driving Mechanisms of Water Environmental Factors on Heavy Metals

**Figure 5:**
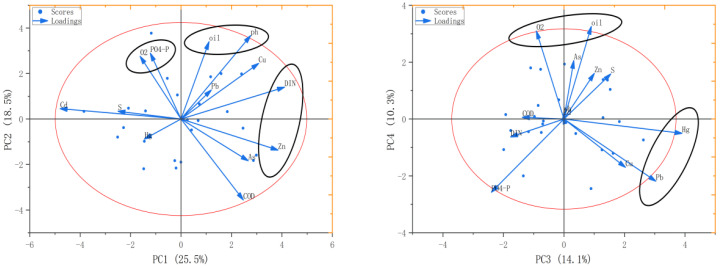
Principal component analysis of heavy metals and environmental factors in the water column of the northern Jiangsu sea area (the first four principal components). Red indicates the confidence ellipse, black represents the cluster area, and orange denotes the load range.

The spatial distribution of dissolved heavy metals in marine systems is jointly influenced by various environmental factors, including pH, DO, oil, phosphate (PO4-P), sulfide (S), and inorganic nitrogen (DIN). To clarify the relationship between heavy metal elements and environmental factors in the study area waters, PCA was performed on six water heavy metals (Cu, Pb, Zn, Cd, Hg, and As) and six water environmental factors (oil, S, PO_4_-P, DIN, DO, and COD). The first six principal components (eigenvalues > 1), accounting for 82.2% of the total variance, were selected. The first four principal components are shown in Figure 5. The first principal component (PC1) accounted for 25.5% of the variance and was dominated by Zn, with DIN being the main driving factor for Zn. The second principal component (PC2) accounted for 18.5% of the variance, with high loadings on pH, oil, PO_4_-P, and DO, indicating that PC2 was dominated by DO. The third principal component (PC3) accounted for 14.1% of the variance and was dominated by Hg and Pb, with both being closely related to PO_4_-P. The fourth principal component accounted for 10.3% of the variance and was dominated by As, with DO and oil content being its main driving factors. Principal components 5 and 6 were dominated by Pb and As, respectively. Sulfide, COD, and PO_4_-P had high loadings in the fifth and sixth principal components, indicating that sulfide content influenced the distribution of Hg and that COD and PO_4_-P were the main driving factors for As. Thus, DIN, PO_4_-P, and oil content are key influencing factors for dissolved heavy metals.

**Figure 6:**
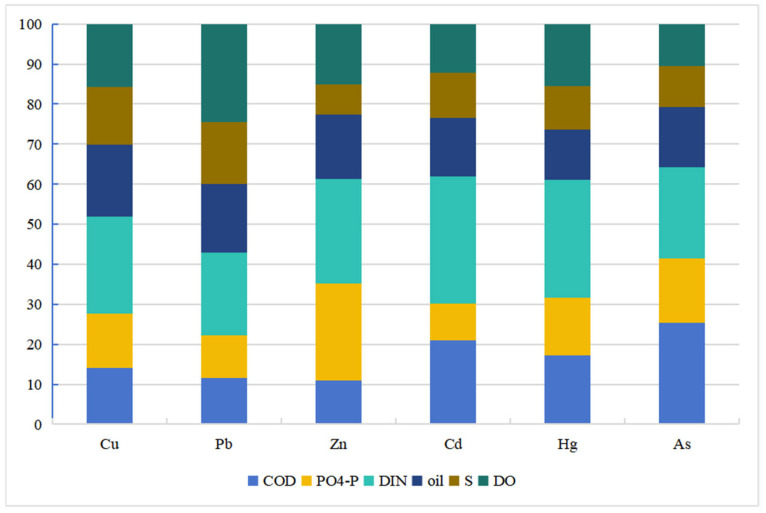
Distributions of environmental factors across the heavy metals.

However, traditional linear statistical methods have difficulty accurately quantifying the relative importance and specific influence characteristics of each factor. As an ensemble learning algorithm, random forest (RF) has strong capabilities for fitting nonlinear relationships, resisting overfitting, and evaluating feature importance, which can effectively identify the differences in the contributions of dominant factors in complex environmental systems [25]. To clarify the core regulatory mechanisms of the contents of six typical heavy metals (As, Cd, Cu, Hg, Pb, and Zn) in environmental media, this study systematically analyzed the environmental regulation characteristics of each heavy metal based on feature importance ranking analysis using the Random Forest model (Figure 6). The decision tree has 200 nodes, a maximum depth of four, and six environmental factors selected as features, with all other parameters unchanged. The results are as follows:

Dissolved inorganic nitrogen (DIN) exhibited a consistently dominant role in influencing heavy metal dynamics. Specifically, DIN was the primary factor for As, Cd, and Zn; the absolute dominant factor for Hg; and a secondary factor for Cu and Pb, although its contribution remained substantially higher than that of other non-core factors. These results indicate that nitrogen nutrients play a fundamental regulatory role in the migration, transformation, and speciation of heavy metals. The secondary core factors for As, Cd, and Hg were all COD, reflecting that organic matter can synergistically regulate the contents of these three heavy metals by altering the redox conditions and complexation capacity of the environmental medium [43]. The secondary core factor for Cu was oil, as oily substances can promote Cu enrichment through interfacial adsorption and hydrophobic effects [43]. The secondary core factor for Pb was DO, highlighting the key influence of a redox environment on Pb speciation transformation and content [44]. The secondary core factor for Zn was PO_4_-P, as phosphorus nutrients can inhibit Zn release by competing for adsorption sites [45]. This reflects the differentiated interaction mechanisms between different heavy metals and environmental factors.

The results of the principal component analysis (PCA) and the random forest (RF) model complemented and validated each other, clarifying the core environmental factors governing the distribution of heavy metals in the waters of the northern Jiangsu coastal area. Dissolved inorganic nitrogen (DIN) emerged as the key environmental factor. In PCA, DIN showed a significant linear correlation with Zn in the first principal component (PC1), acting as the core factor driving Zn distribution. Concurrently, DIN, through interaction with other factors in the fifth and sixth principal components (PC5 and PC6), also jointly influenced As and Cd. The random forest model further quantified this common regulatory role: DIN was the primary core factor for As, Cd, and Zn; it was the absolute dominant factor for Hg; as a secondary core factor for Cu and Pb, its contribution was significantly higher than other non-core factors. This indicates that DIN plays a fundamental regulatory role in the migration, transformation, and speciation of most heavy metals in the water column.

Dissolved oxygen (DO), phosphate phosphorus (PO_4_-P), oil, and chemical oxygen demand (COD) were identified as secondary factors. In the PCA, O_2_ had the highest loading in the second principal component (PC2), with pH, oil, and PO_4_-P jointly influencing the speciation transformation of Cu and Pb. Random forest further specified O_2_ as the secondary core factor for Pb. In the PCA, PO_4_-P showed linear associations with Hg and Pb in the third principal component (PC3) and interacted with DO in PC2. Random forest identified PO_4_-P as the secondary core factor for Zn. In the PCA, oil was associated with Cu in PC2 and with As in the fourth principal component (PC4). Random Forest determined oil to be the secondary core factor for Cu. In the PCA, COD was indirectly associated with As and Hg in PC5 and PC6, while random forest designated COD as the secondary core factor for As, Cd, and Hg. Furthermore, sulfide had only a weak influence on Hg in PC5 of the PCA, suggesting that their direct regulatory effects on water-borne heavy metals are relatively minor.

#### 3.3.2. Source Apportionment of Heavy Metal Pollution in Sediment

**Figure 7:**
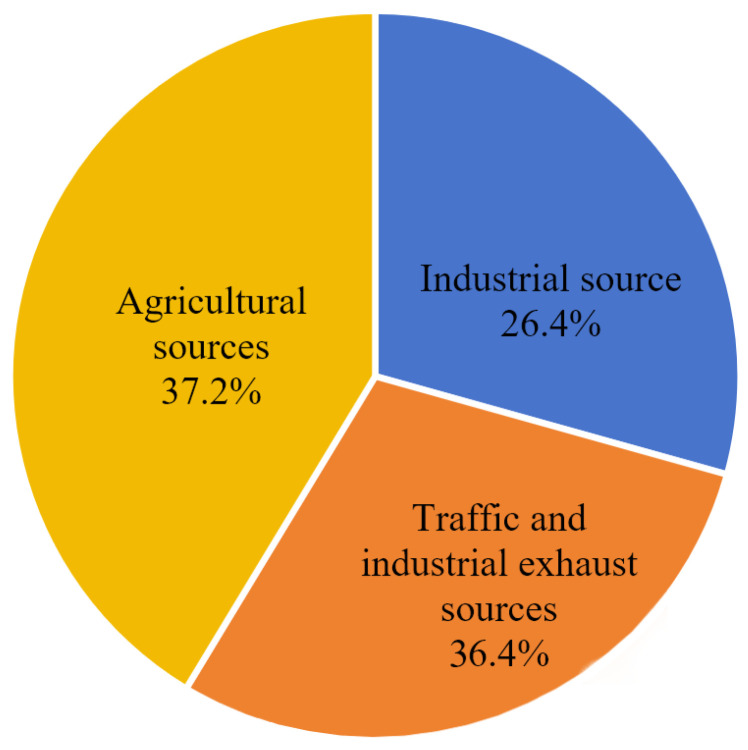
Contribution rate of heavy metal source factors in sediment.

To clarify the pollution sources and contribution degrees of heavy metals in the sediment of the study area, the PMF model was used for source apportionment and contribution rate calculation of seven heavy metals (Cu, Pb, Zn, Cd, Cr, Hg, and As) in sediment. The Q values for each component were between −3 and 3 and showed a normal distribution. PCA was used to further validate the PMF source apportionment results, ultimately obtaining the heavy metal loadings and source distribution in sediment (Figure 7).

**Figure 8:**
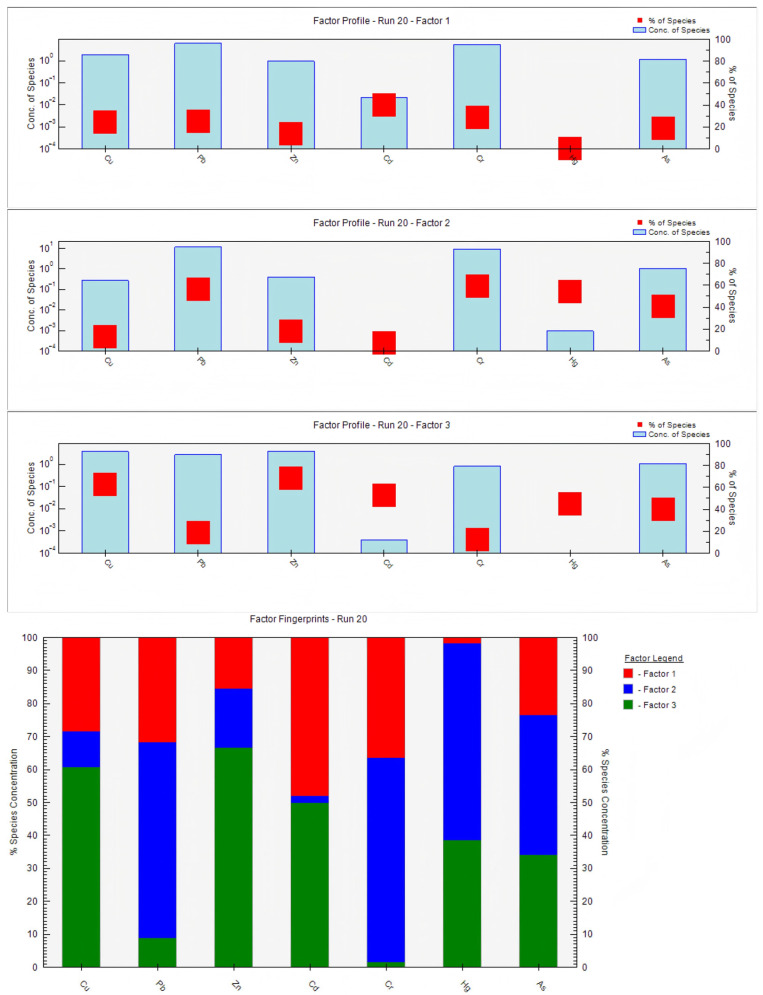
Source-specific loadings and distribution of heavy metals in sediments: pollution source load and pollution source distribution.

The results indicate that sediment heavy metals mainly originate from three types of pollution sources (Figure 8). Factor 1 had a total contribution rate of 26.4%, with significant proportions for Cd (47.9%), Cr (36.5%), and Pb (31.7%). According to marine pollutant statistics, these heavy metals mainly originate from terrestrial industrial wastewater discharge. The study area is adjacent to the new chemical park in Lianyungang Xuwei and the Yanwei Port chemical concentration area, which host heavy-polluting industries such as those for non-ferrous metal smelting, chemicals, and electroplating. In 2020, the industrial wastewater discharge volume in Lianyungang city reached 28.7147 million tons. Even after treatment, industrial wastewater discharged by enterprises may still carry trace amounts of heavy metals like Cd, Cr, and Pb, entering the sea directly through discharge outlets or converging into the study area via rivers like the Guan River and Xinyi River [46,47], consistent with the high contribution of industrial sources to specific heavy metals in the PMF source apportionment. Therefore, Factor 1 is identified as an industrial pollution source.

Factor 2 had a total contribution rate of 36.4%, with significant proportions for Pb (59.5%), Cr (61.9%), Hg (59.7%), and As (42.6%). The accumulation of these elements mainly originates from coal combustion, exhaust emissions, and industrial waste gases. The study area is close to the important port of Yanwei Port, where heavy oil burned by cargo ships and fishing vessels contains large amounts of heavy metals like Pb and Hg. Exhaust gases enter the sea via atmospheric diffusion and dry/wet deposition. To the west, the Lianyungang chemical industrial park and Yancheng Binhai industrial zone house steel and non-ferrous metal smelting enterprises. In 2020, their industrial exhaust emissions contained 6850.89 tons of nitrogen oxides and 2519.45 tons of volatile organic compounds [48], which were transported and deposited into the eastern sea area via atmospheric circulation. Furthermore, the daily traffic volume on the adjacent G15 Shenhai expressway remained high. In 2020, motor vehicle emissions in Lianyungang city reached 15,592.04 tons of nitrogen oxides and 4334.23 tons of volatile organic compounds. Residual heavy metals like Pb in exhaust gases participate in sediment accumulation via atmospheric deposition [49]. These combined inputs from traffic and industrial exhaust are highly consistent with the high loading characteristics of this source for heavy metals (e.g., Pb, Cr, and Hg). Therefore, Factor 2 is identified as a traffic and industrial exhaust source.

Factor 3 had a total contribution rate of 37.2%, with significant proportions for Cu (60.7%) and Zn (66.7%). Previous studies indicate that Cu and Zn mainly originate from pesticides and compound fertilizers. The study area is adjacent to the main agricultural production area of the north Jiangsu plain. As an important commercial grain base in China, this region has vast farmland and intensive agricultural activities. Agricultural runoff from surrounding cities like Lianyungang and Yancheng is an important carrier of pollutants entering the sea. In 2020, the total fertilizer use in Jiangsu Province was controlled below 2.9 million tons, but the average fertilizer use per unit area in the Guan River region of northern Jiangsu still reached 52,500 kg/km^2^, far exceeding the national average [50]. Livestock and poultry farming manure also enriches Cu and Zn. These pollutants enter the sea via surface runoff and agricultural drainage through rivers like the Guan River and Xinyi River, settling into sediment with suspended particles [51,52]. Simultaneously, excessive application of nitrogen and phosphorus fertilizers can form complexes with heavy metals, altering their migration forms and promoting enrichment in sediment, which aligns with the high contribution of agricultural sources to Cu and Zn and the agricultural background of the study area. Therefore, Factor 3 is identified as an agricultural pollution source.

In summary, sediment heavy metal pollution in the study area is jointly driven by agricultural sources (37.2%), traffic and industrial exhaust sources (36.4%), and industrial sources (26.4%). Agricultural sources contribute significantly to Cu and Zn; traffic and industrial exhaust sources contribute greatly to Pb, Cr, and Hg; industrial sources contribute prominently to Cd, Cr, and Pb. The contributions of agricultural, traffic, and industrial exhaust sources are comparable, as they are the main influencing factors of pollution.

Based on the contribution rates of different sources for heavy metals in sediment, the source distribution map for the seven heavy metals in sediment was obtained (Figure 8). The results show that Cu mainly originates from agricultural sources, with a small part from industrial sources. Pb mainly originates from traffic and industrial exhaust, with a small part from industrial activities. Zn mainly originates from agricultural sources. Cd mainly originates from agricultural and industrial pollution. Cr mainly originates from traffic and industrial activities. Hg mainly originates from traffic and industrial exhaust, with a small part from agriculture. As has comparable proportions among the three pollution sources.

### 3.4. Partitioning of Heavy Metals at the Sediment–Water Interface

**Figure 9:**
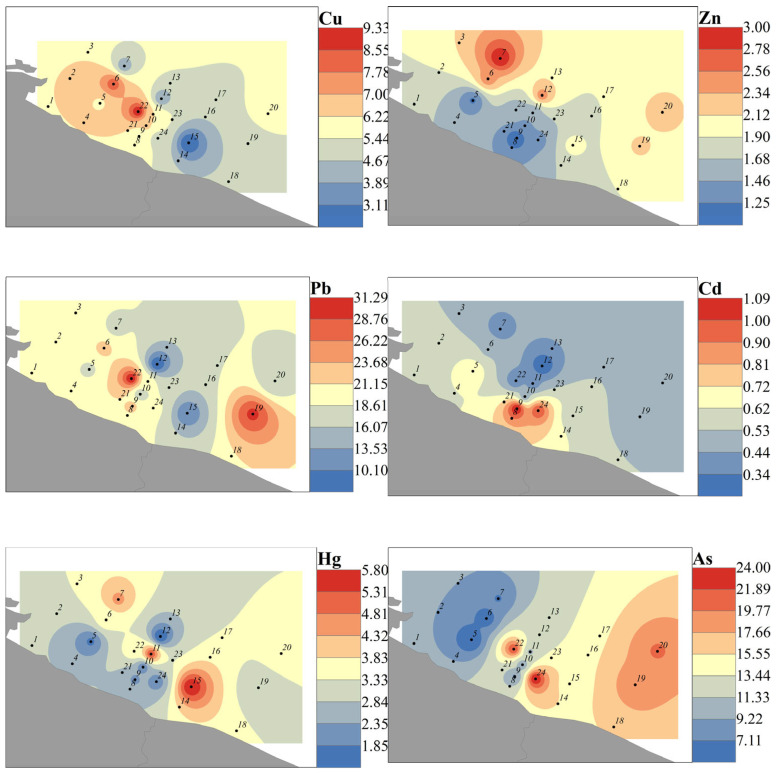
Distributive coefficient of heavy metals at the sediment–water interface in the northern Jiangsu sea area (Yellow Sea). Gray indicates land, and numbers represent sampling locations and order.

The partition coefficient (Kp) of heavy metal elements between different media can reflect their adsorption capacity and migration process. A higher Kp indicates stronger adsorption capacity of sediment for the heavy metal, while a Kp less than one indicates that the heavy metal is more prone to dissolve in water [17]. The calculated Kp results for the six heavy metals in the northern Jiangsu coastal waters are presented in Figure 9. Pb had the highest Kp (average: 19.28 L/g), while Cd had the lowest value (average: 0.55 L/g). The Kp order for the six heavy metals from high to low was as follows: Pb, As, Cu, Hg, Zn, and Cd. Pb, As, and Cu are easily absorbed by particles and deposited in sediment, while Hg, Zn, and Cd are more soluble in water. High Kp values for Cu were mainly distributed in the northwestern sea area. The Kp for Zn gradually increased from the coast towards the open sea. The Kp for Pb showed low values in the central sea area and high values in the surrounding areas. The Kp for Cd gradually decreased from the coast towards the open sea. High Kp values for Hg and As were mainly in the central and southeastern parts of the study area.

Kp calculation results indicated that Pb, As, and Cu possess relatively high Kp values, suggesting their stronger tendency to be adsorbed onto particles and accumulate in sediments, resulting in lower mobility and reduced potential for secondary release. In contrast, Cd, Hg, and Zn exhibited Kp values less than one, indicating a greater propensity to remain in the dissolved phase within the water column, which translates to higher mobility and bioavailability, and, consequently, higher associated ecological risks. This general partitioning pattern is closely linked to the inherent physicochemical properties of each element.

### 3.5. Partitioning of Heavy Metals from Different Sources

**Figure 10:**
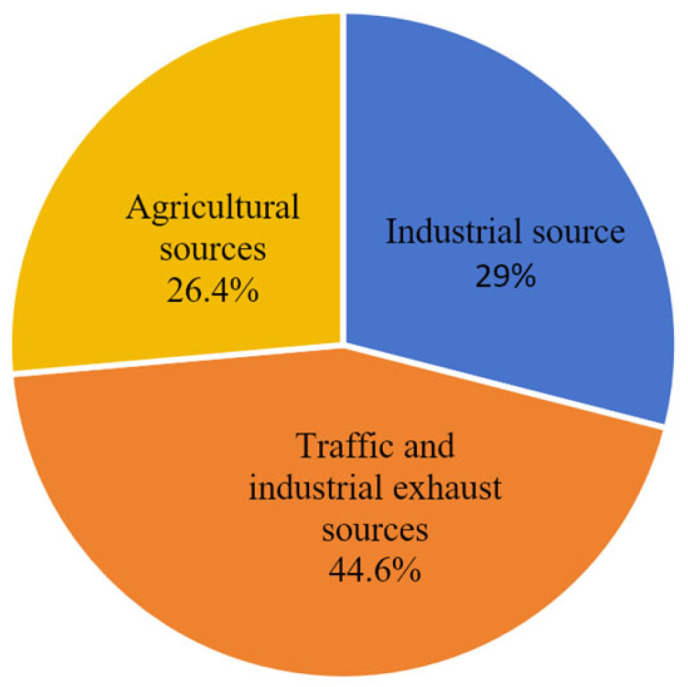
Contribution rates of heavy metal source factors in water bodies.

To better study the partitioning of pollutants from different pollution sources, PMF was performed on heavy metals in water based on the source apportionment results and the partitioning characteristics of heavy metals at the sediment–water interface. Three pollution source proportions for heavy metals in water were obtained: industrial (29%), traffic and industrial exhaust (44.6%), and agricultural (26.4%) (Figure 10). For Cu, the contribution rates of the three sources were 28.6%, 52.8%, and 18.6%, respectively. For Pb, they were 5.2%, 71.4%, and 23.4%. For Zn, they were 48.6%, 49.2%, and 2.2%. For Cd, they were 30.3%, 5.3%, and 64.4%. For Hg, they were 24.3%, 41.7%, and 34%. For As, they were 42%, 45.2%, and 12.7%.

Based on the S-Kp, the partitioning behavior of the six heavy metals from different sources in the study area showed significant source-specific differences (Table 5):

Industrial-Source Heavy Metals: Pb from this source was almost entirely allocated to the sediment phase (S-Kp = 332). Cu and As also showed a strong preference for the sediment phase. In contrast, Cd, Hg, and Zn from industrial sources exhibited a greater tendency to remain in the aqueous phase. This may be attributed to the complex ligand environment present in some industrial wastewaters, which can stabilize certain metals in solutions.

Traffic and Industrial Exhaust-Source Heavy Metals: Pb from atmospheric deposition still predominantly partitioned into sediments (S-Kp = 43.39), suggesting that it is primarily associated with insoluble particulate matter. However, Cu, Zn, Cd, Hg, and As from this source were largely retained in the water phase, indicating that these metals in aerosols may exist as more soluble salts or fine/nanoparticles that readily dissolve upon deposition.

Agricultural-Source Heavy Metals: Cu, Pb, Zn, and As from agricultural runoff were largely distributed to the sediment phase (S-Kp > 10), consistent with the process of sedimentation alongside soil particles and organic matter carried by runoff. Conversely, Cd and Hg from this source remained primarily in the aqueous phase, suggesting that agricultural inputs like certain phosphate fertilizers (which can contain Cd) or historical pesticide residues (containing Hg) may introduce these elements in more soluble or mobile forms.

These findings demonstrate that the environmental fate, speciation, and migration pathways of a given heavy metal can differ fundamentally depending on its original source. Therefore, environmental behavior and risk cannot be assessed based on total element concentration alone; the source must be considered. For instance, Cd originating from industrial sources, despite potentially lower total inputs, may pose a greater ecological threat due to its higher dissolved fraction.

## 4. Conclusions

This study focused on the eastern coastal waters of Yanwei Port in northern Jiangsu. Through the systematic collection of water and sediment samples and the integrated application of principal component analysis (PCA), random forest (RF), positive matrix factorization (PMF) models, the partition coefficient (Kp), and the source-specific partition coefficient (S-Kp), an in-depth investigation was conducted on the distribution characteristics, pollution sources, and partitioning behavior of seven heavy metals (Cu, Pb, Zn, Cd, Cr, Hg, and As) at the water–sediment interface.

1. The study area exhibits a low overall heavy metal pollution risk, but with localized accumulation and specific element concerns. The contents of heavy metals in water all complied with the national Class III seawater quality standards. Assessment using the Risk Quotient (RQ) indicated an overall low-to-moderate ecological risk level. Sediment heavy metal concentrations were generally below the Class I sediment quality standards. The geo-accumulation index (Igeo) revealed only slight to moderate enrichment of As, identifying it as the primary element of concern, while other elements showed no significant anthropogenic enrichment. Spatially, elements with strong spatial variability (e.g., Cd and Pb) and elements with moderate spatial variability (e.g., Hg) exhibited significant local heterogeneity, suggesting the influence of point sources or specific human activities.
2. Heavy metal distribution is significantly regulated by environmental factors, with dissolved inorganic nitrogen (DIN) playing a common dominant role. Combined PCA and random forest models revealed that the distribution of dissolved heavy metals in water is complexly influenced by multiple environmental factors. Among these, DIN demonstrated a common, core regulatory role across different heavy metals. It was the primary factor driving the distribution of Zn, As, and Cd and also significantly influenced the occurrence of Hg, Cu, and Pb. Dissolved oxygen (DO), phosphate phosphorus (PO_4_-P), oil content, and chemical oxygen demand (COD) acted as secondary factors, with their influence showing distinct heavy metal specificity.
3. Sediment heavy metals originate from a typical “industry-agriculture-traffic” composite pollution pattern. PMF source apportionment results indicated that sediment heavy metals primarily stem from three pollution sources with comparable contributions: agricultural sources, traffic and industrial exhaust sources, and industrial sources. Agricultural sources were the main contributors to Cu and Zn. Traffic and industrial exhaust sources dominated the inputs of Pb, Cr, and Hg, highlighting the importance of atmospheric deposition pathways. Industrial sources made prominent contributions to Cd, Cr, and Pb, reflecting the influence of adjacent industrial zones.
4. The partitioning behavior of heavy metals at the sediment–water interface shows significant source-specific differences. The overall partition coefficients (Kp) indicated that Pb, As, and Cu are more readily adsorbed and immobilized in sediments, while Cd, Hg, and Zn tend to remain in the aqueous phase, possessing higher mobility and potential bioavailability. More importantly, S-Kp analysis revealed that the partitioning behavior of the same heavy metal element between the two phases differs fundamentally depending on its source. For example, Pb derived from industrial sources was almost entirely allocated to sediment, while traffic and industrial exhaust-source Cu and Zn were mainly distributed in the water column. Agricultural-source Cu, Pb, Zn, and As were prone to settle with particulates, yet the Cd and Hg inputs from this source remained higher in the aqueous phase. This unveils the new insight that the “pollution source determines environmental fate”.

This study, by coupling multivariate statistical models (PCA, RF, and PMF) with physico-chemical partitioning parameters (Kp and S-Kp), achieved a holistic chain analysis from “distribution characteristics–driving factors–pollution source tracing–interfacial behavior”. The findings not only clarify the major sources and key migration processes of heavy metals in the northern Jiangsu coastal waters but also, more importantly, by identifying the differential partitioning behaviors of heavy metals from different sources, offer direct scientific support for implementing precise “source-pathway-receptor” management and formulating differentiated pollution prevention and control strategies. This holds substantial practical value for ensuring the safety of aquaculture and the health of the nearshore ecosystem in this region.

## Figures and Tables

**Table 1:** Statistical table of heavy metal concentrations in water bodies.

Statistic	Cu	Pb	Zn	Cd	Hg	As
Maximum/(μg/L)	2.1	0.82	19	0.37	0.028	1.4
Minimum/(μg/L)	1.2	0.23	11	0.07	0.01	0.8
Standard deviation/(μg/L)	1.73	0.49	15.3	0.18	0.019	1.04
Coefficient of variation (%)	12.1	31.85	16.5	43.87	26.11	15.65

**Table 2:** Statistical table of heavy metals in waters from different sea areas.

Maritime Space	Year	Cu (μg/L)	Pb (μg/L)	Zn (μg/L)	Cd (μg/L)	Hg (μg/L)	Data Source
This Research	2021	1.73	0.49	15.83	0.19	0.021	This study
Pearl River Estuary	2018	2.56	0.82	22.1	0.31	0.035	[33]
Hormuz	2022	4.8	1.5	42.3	0.45	0.058	[34]
Chesapeake Bay	2021	1.5	0.45	14.7	0.18	0.020	[35]
Coast of Korea	2021	3	0.19	26.2	0.19	0.018	[36]

**Table 3:** Statistical table of heavy metal content in sediment.

Statistic	Cu	Pb	Zn	Cd	Cr	Hg	As
Maximum/(mg/kg)	12.25	34.5	13.8	0.12	28	0.074	9.8
Minimum/(mg/kg)	4.9	19.5	5.4	0.04	14.4	0.032	6.2
Standard deviation/(mg/kg)	9.42	26.25	8.75	0.09	20.8	0.053	7.5
Coefficient of variation (%)	24.24	22.19	29.42	28.58	23.34	27.56	15.45

**Table 4:** Statistical table of heavy metals in sediments from different sea areas.

Maritime Space	Year	Cu (mg/kg)	Pb (mg/kg)	Zn (mg/kg)	Cd (mg/kg)	Cr (mg/kg)	Data Source
This Research	2021	9.42	26.25	8.75	0.09	20.8	This study
Indian coastal	2020	43.35	51.92	145.54	/	79.35	[42]
Mediterranean coastal	2020	25.91	15.47	45.84	/	32.6	[41]
East China Sea	2015	25	24.8	79.1	0.096	/	[40]

**Table 5:** Distribution coefficient of heavy metals from different sources.

Factors	Cu	Pb	Zn	Cd	Hg	As
Industrial source	5.45	332	0.12	0.86	0.18	4
Traffic and industrial sources	1.12	43.39	0.14	0.2	4	6.8
Agricultural sources	17.88	17.77	11.66	0.375	3.3	19.62

## Data Availability

The original contributions presented in this study are included in the article. Further inquiries can be directed to the corresponding author.

## Abbreviations

The following abbreviations are used in this manuscript:

PCA	Principal component analysis
PMF	Positive matrix factorization
RF	Random forest
Kp	Partition coefficient
S-Kp	Source-specific partition coefficient
Cu	Copper
Zn	Zinc
Pb	Plumbum
Cr	Chromium
As	Arsenic
Hg	Mercury
Cd	Cadmium

## References

[1] Masindi V., Mkhonza P., Tekere M., Masindi V., Tekere M. (2021). Sources of heavy metals pollution. Remediation of Heavy Metals.

[2] Zhao Y., Li J., Liu H. (2019). Heavy metal pollution in coastal sediments of the Yellow Sea: A review. Mar. Pollut. Bull..

[3] Angon P.B., Islam M.S., Das A., Anjum N., Poudel A., Suchi S.A. (2024). Sources, effects and present perspectives of heavy metals contamination: Soil, plants and human food chain. Heliyon.

[4] Liao J., Qian X., Liu F., Deng S., Lin H., Liu X., Wei C. (2021). Multiphase distribution and migration characteristics of heavy metals in typical sandy intertidal zones: Insights from solid-liquid partitioning. Ecotoxicol. Environ. Saf..

[5] Wang X., Chen J., Zhang L. (2021). Distribution and partitioning of heavy metals in water-sediment system of a typical coastal wetland in eastern China. Ecotoxicol. Environ. Saf..

[6] Han S.H., Wang C., Liu X.P., Yu Y., Xu M.Q., Shang Q.W. (2021). On distribution characteristics and pollution evaluation of soil heavy metals in rice producing areas of Lianyungang. J. Southwest China Norm. Univ. (Nat. Sci. Ed.).

[7] Gao Q., Wang N., Xiang L., Sui Q. (2025). Distribution and pollution assessment of heavy metals in seawater, surface sediments and marine organisms in Lianyungang offshore, China. Reg. Stud. Mar. Sci..

[8] Li W., Liu F., Zhang W., Wang X. (2025). Spatial distribution, sources, and ecological risk assessment of heavy metals in Lianyungang coastal sediments of China. Environ. Monit. Assess..

[9] Jin M., Yuan H., Liu B., Peng J., Xu L., Yang D. (2020). Review of the distribution and detection methods of heavy metals in the environment. Anal. Methods.

[10] Liu J., Kang H., Tao W., Li H., He D., Ma L., Tan Y., Li X. (2023). A spatial distribution–Principal component analysis (SD-PCA) model to assess pollution of heavy metals in soil. Sci. Total Environ..

[11] Zhiyuan W., Dengfeng W., Huiping Z., Zhiping Q.I. (2011). Assessment of soil heavy metal pollution with principal component analysis and geoaccumulation index. Procedia Environ. Sci..

[12] Chen H., Wu D., Wang Q., Fang L., Wang Y., Zhan C., Liu Y., Liu S., Shen J. (2022). The predominant sources of heavy metals in different types of fugitive dust determined by principal component analysis (PCA) and positive matrix factorization (PMF) modeling in Southeast Hubei: A typical mining and metallurgy area in Central China. Int. J. Environ. Res. Public Health.

[13] Paatero P., Tapper U. (1994). Positive matrix factorization: A non-negative factor model with optimal utilization of error estimates of data values. Environmetrics.

[14] Wang D.K., Xu L.M., Zheng J.L., Bi X., Yao Y., Cai Y., Guo X., Liu J., Tan B. (2025). Analysis of current status and source apportionment of soil heavy metal pollution based on PMF model and Pb isotope tracing. Geol. Bull. China.

[15] Miranda L.S., Wijesiri B., Ayoko G.A., Egodawatta P., Goonetilleke A. (2021). Water-sediment interactions and mobility of heavy metals in aquatic environments. Water Res..

[16] Kong M., Zhu Y., Han T., Zhang S., Li J., Xu X., Gao Y. (2021). Interactions of heavy metal elements across sediment-water interface in Lake Jiaogang. Environ. Pollut..

[17] Zhao H., Chen X.D., Li M. (2023). Application of specific partition coefficient in source apportionment of heavy metals at the water-sediment interface. China Environ. Sci..

[18] (2008). The Specification for Marine Monitoring—Part 3: Sample Collection, Storage and Transportation. General Administration of Quality Supervision, Inspection and Quarantine of the People’s Republic of China, & Standardization Administration of the People’s Republic of China.

[19] (2013). Specification for Ocean Monitoring Technology—Part 1: Sea Water. State Oceanic Administration.

[20] (2013). Specification for Ocean Monitoring Technology—Part 2: Sediment. State Oceanic Administration.

[21] (2007). The Specification for Marine Monitoring—Part 5: Sediment Analysis. National Technical Committee for Marine Standardization (SAC/TC 283).

[22] Pang K., Luo K., Zhang S., Hao L. (2024). Source-oriented health risk assessment of groundwater based on hydrochemistry and two-dimensional Monte Carlo simulation. J. Hazard. Mater..

[23] Men C., Wang Y., Liu R., Wang Q., Miao Y., Jiao L., Shoaib M., Shen Z. (2021). Temporal variations of levels and sources of health risk associated with heavy metals in road dust in Beijing from May 2016 to April 2018. Chemosphere.

[24] Wang N., Wang N., Qi D., Kang G., Wang W., Zhang C., Zhang Z., Zhang Y., Zhang H., Zhang S. (2023). Comprehensive overview of antibiotic distribution, risk and priority: A study of large-scale drinking water sources from the lower Yangtze River. J. Environ. Manag..

[25] Liu T., Wang M., Wang M., Xiong Q., Jia L., Ma W., Guo X. (2025). Identification of the primary pollution sources and dominant influencing factors of soil heavy metals using a random forest model optimized by genetic algorithm coupled with geodetector. Ecotoxicol. Environ. Saf..

[26] Zhang L., Bai J., Zhang K., Zhai Y., Wang Y., Liu H., Xiao R., Jorquera M.A., Xia J. (2023). Spatial variability, source identification and risks assessment of antibiotics in multimedia of North China’s largest freshwater lake using positive matrix factorization and Monte Carlo simulation. J. Hazard. Mater..

[27] Li Q., Gao J., Zhang Q., Liang L., Tao H. (2017). Distribution and risk assessment of antibiotics in a typical river in North China plain. Bull. Environ. Contam. Toxicol..

[28] Day J.W., Rivera-Arriaga E., del Carmen Peña-Puch A., Hunter R.G. (2024). Sustainability of Gulf of Mexico coastal estuaries and lagoons: Interactions with hydrocarbon production—A review with a look to the future. Sustainability.

[29] Lin Z., Li J., Luan Y., Dai W. (2020). Application of algae for heavy metal adsorption: A 20-year meta-analysis. Ecotoxicol. Environ. Saf..

[30] Zhang R., Zhang F., Ding Y., Gao J., Chen J., Zhou L. (2013). Historical trends in the anthropogenic heavy metal levels in the tidal flat sediments of Lianyungang, China. J. Environ. Sci..

[31] (1997). Sea Water Quality Standard. State Oceanic Administration.

[32] Wheeler J.R., Grist E.P.M., Leung K.M.Y., Morritt D., Crane M. (2002). Species sensitivity distributions: Data and model choice. Mar. Pollut. Bull..

[33] Li P., Guo X., Yang Q., Luo X., Liu F., Dong H., Tan C. (2017). Current status of heavy metal pollution in nearshore waters of the Pearl River Estuary. Mar. Environ. Sci..

[34] Ebrahimi-Sirizi Z., Riyahi-Bakhtiyari A., Ghasempouri S.M. (2022). Heavy metals in Persian Gulf coastal waters: Pollution, sources, and ecological risk assessment. Mar. Pollut. Bull..

[35] U.S. Environmental Protection Agency (USEPA) (2022). Chesapeake Bay Water Quality Monitoring Program 2021.

[36] Hwang K., Lee J., Kwon I., Park S.Y., Yoon S.J., Lee J., Khim J.S. (2021). Large-scale sediment toxicity assessment over the 15,000 km of coastline in the Yellow and Bohai seas, East Asia. Sci. Total Environ..

[37] (2002). Marine Sediment Quality. National Center of Ocean Standards and Metrology.

[38] Zaakour F., Kholaiq M., Khouchlaa A., El Mjiri I., Rahimi A., Saber N. (2023). Assessment of heavy metal contamination using pollution index, geo-accumulation index, and potential ecological risk index in agricultural soil–A case study in the coastal area of Doukkala (Morocco). Ecol. Eng. Environ. Technol..

[39] Wedepohl K.H. (1995). The composition of the continental crust. Geochim. Cosmochim. Acta.

[40] Wang R., Zhang C., Huang X., Zhao L., Yang S., Struck U., Yin D. (2020). Distribution and source of heavy metals in the sediments of the coastal East China sea: Geochemical controls and typhoon impact. Environ. Pollut..

[41] Abbasi A., Mirekhtiary F. (2020). Heavy metals and natural radioactivity concentration in sediments of the Mediterranean Sea coast. Mar. Pollut. Bull..

[42] Ramasamy V., Senthil S., Paramasivam K., Suresh G. (2022). Potential toxicity of heavy metals in beach and intertidal sediments: A comparative study. Acta Ecol. Sin..

[43] Li Y., Gong X. (2021). Effects of dissolved organic matter on the bioavailability of heavy metals during microbial dissimilatory iron reduction: A review. Rev. Environ. Contam. Toxicol..

[44] Zhao S., Feng C., Wang D., Liu Y., Shen Z. (2013). Salinity increases the mobility of Cd, Cu, Mn, and Pb in the sediments of Yangtze Estuary: Relative role of sediments’ properties and metal speciation. Chemosphere.

[45] Mphuthi B.R. (2021). Assessing the Pollutant Removal Efficiency of a Wetland as a Polishing Treatment for Municipal Wastewater. Master’s Thesis.

[46] Liu Z., Fei Y., Shi H., Mo L., Qi J. (2022). Prediction of high-risk areas of soil heavy metal pollution with multiple factors on a large scale in industrial agglomeration areas. Sci. Total Environ..

[47] Jiao Y.F., Wang Y. (2018). Study on heavy metal content in soil of chemical industry zone in Lianyungang City. Sci. Technol. Vis..

[48] Han R., Luo H., Leng H., Wu W., Chen L., Liu M., He B.J. (2025). Risk assessment of heavy metals in road dust and simulation of pollutant release in Lianyungang city. Chem. Ecol..

[49] Abderrahmane B., Naima B., Tarek M., Abdelghani M. (2021). Influence of highway traffic on contamination of roadside soil with heavy metals. Civ. Eng. J..

[50] Yuan L., Liu Q., Jian H., Mi T., Yang F., Yao Q. (2025). Spatial and temporal distribution of metal elements in the rivers of Northern Jiangsu Province. J. Ocean Univ. China.

[51] Wan Y., Liu J., Zhuang Z., Wang Q., Li H. (2024). Heavy metals in agricultural soils: Sources, influencing factors, and remediation strategies. Toxics.

[52] Salem M.A., Bedade D.K., Al-Ethawi L., Al-Waleed S.M. (2020). Assessment of physiochemical properties and concentration of heavy metals in agricultural soils fertilized with chemical fertilizers. Heliyon.

